# Empirical Tuberculosis Treatment in Human Immunodeficiency Virus (HIV)-Associated Fever of Unknown Origin: A Case-Based Rationale

**DOI:** 10.7759/cureus.87769

**Published:** 2025-07-12

**Authors:** Matthew Antonioli, Samantha H Antonioli, Gabriel M Aisenberg

**Affiliations:** 1 Internal Medicine, University of Texas Health Science Center at Houston, Houston, USA

**Keywords:** acquired immune deficiency syndrome, fever of unknown origin, human immunodeficiency virus, interferon gamma release assay, lipoarabinomannan, tuberculosis

## Abstract

Fever of unknown origin (FUO) in people living with human immunodeficiency virus (PLHIV) is clinically defined as recurrent fever lasting more than four weeks in the outpatient setting or more than three days during hospitalization, despite a thorough diagnostic evaluation. This evaluation typically includes a comprehensive medical history, physical examination, imaging studies (such as chest radiography), and an extensive range of laboratory tests, including complete blood counts, blood and urine cultures, and metabolic panels.

Among the many possible causes, tuberculosis (TB) stands out as a leading concern, given its disproportionate burden in PLHIV. However, whether to initiate empirical anti-tubercular therapy in HIV-positive patients presenting with FUO remains a matter of clinical debate. The variability in regional TB prevalence and resource availability makes universal recommendations difficult to apply.

In this context, we review the available evidence supporting the use of empirical TB treatment in PLHIV with FUO, aiming to guide clinical decision-making in settings where diagnostic certainty is elusive.

## Introduction

Tuberculosis (TB) remains a significant global health burden, accounting for approximately 10 million new cases and an estimated 1.2 million deaths annually, with a disproportionate impact on individuals living with human immunodeficiency virus (PLHIV) [1]. The prevalence of TB-HIV coinfection varies geographically, reflecting differences in epidemiology and healthcare infrastructure.

Fever of unknown origin (FUO) in PLHIV is clinically defined as recurrent fever lasting more than four weeks in an outpatient setting or more than three days in an inpatient setting, despite a comprehensive diagnostic evaluation. This workup typically includes detailed medical history, physical examinations, imaging studies (e.g., chest radiography), and a broad array of laboratory investigations such as complete blood counts, blood and urine cultures, and metabolic panels.

The differential diagnosis of FUO in PLHIV is broad and encompasses not only TB but also a wide range of opportunistic infections, including viral, bacterial, fungal, and parasitic etiologies, as well as malignancies [2]. In the general population, non-infectious conditions are increasingly recognized as causes of fever of unknown origin (FUO). However, in PLHIV, particularly those not receiving antiretroviral therapy, infections remain the predominant cause [3]. When evaluating FUO in this population, key considerations include the degree of immunodeficiency and regional epidemiological factors, such as the local prevalence of specific infections [4,5]. 

The decision to initiate empirical anti-tubercular therapy in HIV-positive individuals presenting with FUO remains a topic of clinical debate, and it is backed up by a variety of circumstances or diagnostic interventions. Some of them are summarized and tabulated in this article centered around a case presentation.

## Case presentation

**Figure 1:**
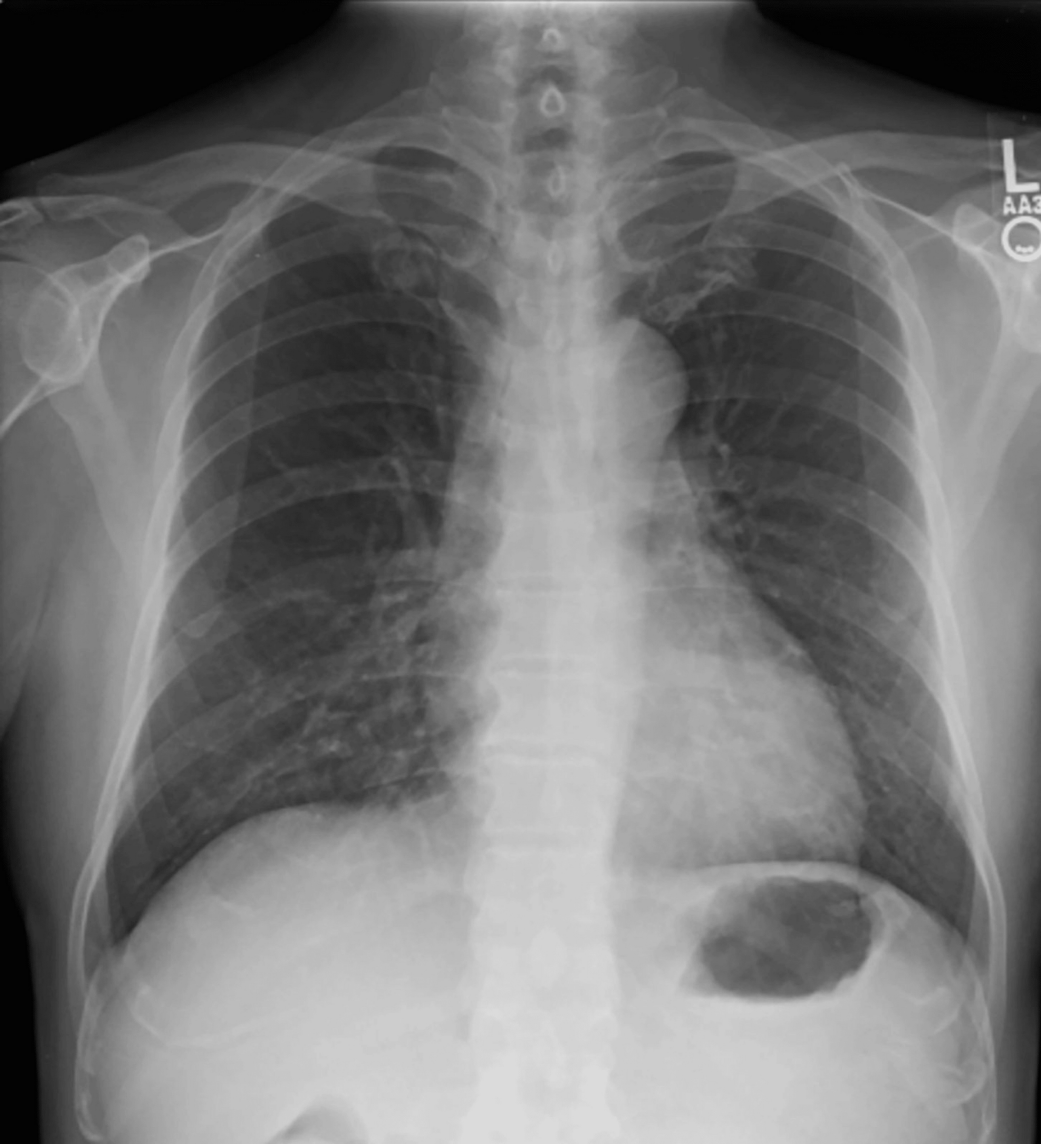
Chest radiograph Chest radiography demonstrating bilateral perihilar interstitial infiltrates

A 57-year-old Vietnamese male presented with a one-month history of fever and fatigue and was subsequently diagnosed with HIV-infection-associated acquired immunodeficiency syndrome. Chest radiography demonstrated bilateral perihilar interstitial infiltrates (Figure 1).

Sputum analysis confirmed the presence of *Pneumocystis jirovecii* (PJP) via direct fluorescence antibody testing. During this admission, laboratory results showed a hemoglobin level of 9.4 g/dL with a normal mean corpuscular volume, normal white blood cell count and platelets, and a low serum sodium that normalized rapidly. *Legionella* and *pneumococcus* urine antigens and cryptococcal serum antigen were negative. The patient was treated with trimethoprim-sulfamethoxazole and prednisone, resulting in clinical improvement, and was discharged.

**Figure 2:**
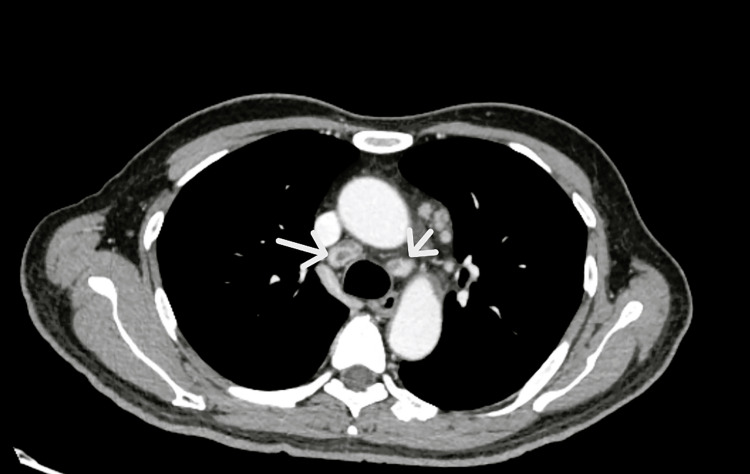
Chest computed tomography Chest computed tomography of the chest revealed necrotic-appearing, enlarged mediastinal lymph nodes (white arrows) with otherwise normal lung parenchyma.

Ten days post-discharge, while receiving antiretroviral therapy (emtricitabine, tenofovir alafenamide, and bictegravir) alongside ongoing treatment for PJP, the patient was readmitted with fever and systemic symptoms. Laboratory testing revealed an increased CD4+ count and a marked reduction in viral load. Despite these improvements, febrile episodes persisted. Chest radiography was unchanged from previous imaging; however, computed tomography of the chest, abdomen, and pelvis revealed necrotic-appearing, enlarged mediastinal lymph nodes with otherwise normal lung parenchyma (Figure 2).

Relevant laboratory data are presented in Table 1. Liver and kidney function tests were normal.

**Table 1:** Patient’s laboratory results

Patient’s laboratory results		
Test	Result	Reference values
Hemoglobin	9.6 g/dL	13-15 g/dL
Mean corpuscular volume (MCV)	90.5 mm^3^	88-92 mm^3^
White cell count	7,300 cells/mm^3^	5,000-10,000 cells/mm^3^
Platelet count	228,000/mm^3^	150,000-300,000/mm^3^
HIV viral load		
First admission	1,680,000 copies/mL	<20 copies/mL
Second admission	1,790 copies/mL	<20 copies/mL
CD4 count		
First admission	4 cells/μL	>500 cells/μL
Second admission	14 cells/μL	>500 cells/μL
SARS-CoV-2 nasal PCR	negative	Negative
Serum antigens:		
Histoplasma capsulatum	negative	negative
Blastomyces species	negative	negative
Cryptococcus neoformans	negative	negative
Serum antibodies		
Aspergillus	negative	negative
Blastomyces species	negative	negative
Serum HHV-6 PCR	negative	negative
Serum JC virus PCR	negative	negative
HSV-1 and 2 virus PCR	negative	negative
Blood cultures (x 3)	negative	negative
Interferon-gamma-release assay for Mycobacterium tuberculosis	positive	negative

Bronchoalveolar lavage (BAL) fluid was negative for *P. jirovecii*, mycobacteria, and fungi. A transbronchial ultrasound-guided lymph node biopsy yielded benign bronchial epithelial cells, lymphoid tissue, and necrotic debris, with no evidence of metastatic carcinoma. Special stains for acid-fast bacilli (AFB) and fungi (*Grocott methenamine silver* [GMS]) were negative.

A bone marrow biopsy was performed to assess normocytic anemia and FUO. Histopathological analysis included staining for iron, CD68, AFB, GMS, reticulin, Masson’s trichrome, and cytomegalovirus (CMV). The marrow showed scattered histiocytes without evidence of malignancy, granulomatous inflammation, or infection by fungi, mycobacteria, or CMV. Notably, cultures grew *Staphylococcus capitis*, which was treated successfully with clindamycin based on antimicrobial susceptibility testing.

The patient had no known history of TB infection or a positive tuberculin skin test. Given the clinical and radiographic findings, empirical anti-tubercular therapy was initiated with rifampin, isoniazid, ethambutol, and pyrazinamide. Antiretroviral therapy was adjusted to emtricitabine, tenofovir, and dolutegravir to mitigate drug-drug interactions with rifampin. The patient's fever resolved within four days of initiating anti-TB therapy, and he was subsequently discharged. Twenty days later, the culture of the mediastinal lymph node confirmed the growth of *Mycobacterium tuberculosis* by high-performance liquid chromatography (HPLC). The isolate was sensitive to isoniazid, rifampin, ethambutol, pyrazinamide, and ofloxacin, by Agar proportion. The patient completed therapy with isoniazid, rifampin, ethambutol, and pyrazinamide. He is currently asymptomatic, has an undetectable HIV viral load, and has a CD4 count of 143/ml (8%).

## Discussion

Table 2 provides a summary of the current evidence supporting the use of empirical TB therapy in PLHIV [6-18].

**Table 2:** Summary of the current evidence supporting the use of empirical tuberculosis therapy in people living with HIV

Type of Diagnosis	Evidence to support the test
Epidemiology	A high prevalence lowers the negative predictive value of screening tests. In areas of high prevalence, the decision to treat empirically is frequently based on the need for rapid symptom relief, limited resources for repeat testing, the patient’s capacity to provide a reliable sputum sample, and antibiotic and testing availability [6].
Clinical Presentation	Current cough, fever, night sweats, and unintentional weight loss can be used in resource-limited settings to identify PLHIV who need further diagnostic testing [9]. This approach has a sensitivity of 78% and a specificity of 49% [9]. In Uganda, empiric treatment based on >2 weeks of cough AND World Health Organization (WHO) defined “danger signs” (tachycardia >120 beats/minute, fever >39C, or tachypnea >30 breath/minute) increased patient survival (44% reduction in 8-week mortality) in PLHIV [6].
General Laboratory Data	Moderate to severe anemia (Hb<10.9 g/dL in men and Hb<9.9 g/dL in women) is associated with a high prevalence of undiagnosed pulmonary TB in PLHIV [9]. Sensitivity of screening tests in PLHIV with moderate to severe anemia is improved when compared with patients who had no/mild anemia: Urine lipoarabinomannan (LAM): 54% vs. 0%; sputum Xpert: 74% vs. 41% [9].
Imaging	In patients with CD4 < 200 cells/ml, chest radiograph findings are predominantly non-cavitary infiltrates and consolidation (Infiltration > consolidation > cavity > lymphadenopathy) [10]. Miliary pattern and pericardial effusion significantly increased among patients with CD4 less than 200 cells/mL. Typical TB pulmonary lesions are seen only in about 33% of PLHIV with TB co-infection [10]. In PLHIV with TB, computed tomography (CT) commonly shows lymphadenopathy and nodular opacities with an increased prevalence of miliary disease. Consolidation and cavitation are less frequent [8]. On CT, interlobular septal thickening, necrotic lymph nodes, and extra-thoracic involvement are commonly seen [12].
Lipoarabinomannan (LAM) in urine	A glycolipid of M. tuberculosis’s cell wall is released by metabolically active or degrading bacterial cells. More sensitive with low CD4 counts [13,14]. WHO recommends Alere-LAM to diagnose TB in PLHIV with CD4 count <100 cell/ml; pooled sensitivity and specificity of LF-LAM in patients with CD4 thresholds <100 cells/μL were 61% and 89%, respectively [14] - FujiLAM has shown a sensitivity of 87.1% (95% CI 79.3%-93.6%) [15]. Moderate and severe anemia increases urine LAM sensitivity [9]
Nucleic Acid Amplification Test (NAAT)	In 2010, WHO recommended that Xpert, an NAAT, be used as the initial diagnostic test in PLHIV with suspected TB. It cannot be used as a test of cure because the test remains positive after an infection is cleared [12]. NAAT with a positive AFB smear is 98% sensitive, and with negative smear is 72%, 85%, and 90% sensitive using one, two, or three specimens, respectively [16]. A positive NAAT result in a smear-negative patient can influence early treatment of TB in approximately 50% to 80% of cases [14].
Tuberculin Skin Test (TST)	In Canada, this screening test detected high positive results in Canadian and foreign-born PLHIV should be screened due to a high number of cases [16]. Sensitivity in PLHIV 64.3% with a cutoff of 10 mm, 71.2% with a cutoff of 5 mm [17].
Interferon Gamma Release Assay (IGRA)	Similar value than TST but more reliable in populations with previous BCG vaccine [14]. Its sensitivity is not increased when used in conjunction with the TST. Cannot differentiate between latent tuberculosis infection, active TB, or past TB [14]. Sensitivity is lower in PLHIV (63%) versus HIV negative (84%) [18]

Evaluating and managing FUO in PLHIV poses a distinct clinical challenge, particularly in balancing the potential risks of empirical anti-tubercular treatment, such as hepatotoxicity, against the serious consequences of missing or delaying treatment for active TB. Clinicians must consider multiple factors, including regional TB prevalence, diagnostic test performance characteristics (sensitivity and specificity), and the risk of patient attrition due to loss to follow-up [19].

The decision to withhold empirical therapy is not without its hazards, as PLHIV, especially those with profound immunosuppression and low CD4+ counts, often present atypically, frequently lacking classic clinical or radiographic features of TB [20]. Importantly, deferring anti-TB therapy should not delay the initiation of antiretroviral treatment (ART), as ART itself confers a protective effect against the development of TB in this population [20].

In the case presented, the decision to initiate empirical TB treatment was based on a constellation of clinical factors, including persistent fever, imaging findings suggestive of lymphadenopathy with necrosis, unexplained anemia, and a positive interferon-gamma release assay (IGRA). While the toxicity of anti-TB medications warrants careful consideration, empirical therapy may also obviate the need for further invasive testing and prevent deterioration from undiagnosed TB or coexisting opportunistic infections.

Notably, in critically ill PLHIV, the pre-test probability of active TB, supported by autopsy data, can approach 50%, reinforcing the rationale for early empirical intervention in selected cases [8,9]. In this patient, the cumulative clinical and diagnostic evidence strongly favored the initiation of empirical TB therapy, as outlined in Table 1. Ultimately, the culture of the lymph node confirmed *Mycobacterium tuberculosis*, validating the clinical decision and avoiding potentially harmful delays in treatment.

## Conclusions

The evaluation and management of fever of unknown origin (FUO) in people living with HIV (PLHIV) remain complex and nuanced. Although infectious causes continue to be the most common diagnoses in both HIV-positive and HIV-negative individuals, particularly among those receiving antiretroviral therapy, non-infectious etiologies are being increasingly recognized. However, in PLHIV with advanced immunodeficiency, such as the case presented, infections remain the predominant cause of FUO.

A comprehensive assessment that integrates epidemiological context, clinical findings, laboratory results, and imaging studies is essential to inform diagnostic and therapeutic decisions. This approach must carefully weigh the potential benefits of initiating specific treatments against the risks they may entail.

Given the global diversity in the prevalence of tuberculosis as a cause of FUO, healthcare resources, and clinical expertise, a universally standardized algorithm for FUO in PLHIV is not feasible. Instead, clinical judgment should prevail, and the decision to start empirical anti-tuberculous therapy, among other treatments, must be tailored to the individual patient's presentation and the local epidemiological and healthcare context.
